# Selenium Speciation in Commonly Consumed Thai Seafood Under Different Cooking Methods

**DOI:** 10.3390/foods15122052

**Published:** 2026-06-06

**Authors:** Narisa Rueangsri, Chonnikarn Limpaninchart, Niratchaporn Thanopajai, Kunchit Judprasong, Piyanut Sridonpai, Nunnapus Laitip, Nattikarn Ornthai, Jörg Feldmann, Alongkote Singhato

**Affiliations:** 1 Nutrition and Dietetics Division, Faculty of Allied Health Sciences, Burapha University, Chonburi 20131, Thailand; narisar@go.buu.ac.th (N.R.); chonnikarn.limp@gmail.com (C.L.); niratchaporn.tn@gmail.com (N.T.); 2 Master of Science Program (Biomedical Sciences), Faculty of Allied Health Sciences, Burapha University, Chonburi 20131, Thailand; 3 Institute of Nutrition, Mahidol University, Salaya, Phutthamonthon, Nakhon Pathom 73170, Thailand; kunchit.jud@mahidol.ac.th (K.J.); piyanut.sri@mahidol.ac.th (P.S.); 4 Chemical Metrology and Biometry Department, National Institute of Metrology, Pathum Thani 12120, Thailand; nunnapusl@nimt.or.th (N.L.); nattikarn@nimt.or.th (N.O.); 5 Trace Element Speciation Laboratory (TESLA), Institute of Chemistry, University of Graz, 8010 Graz, Austria; joerg.feldmann@uni-graz.at

**Keywords:** HPLC-ICP-QQQ-MS, selenium, seafood, speciation analysis

## Abstract

Selenium (Se) speciation in seafood is a key determinant of its nutritional value. However, limited data exist on the influence of common cooking methods on Se chemical forms. This study investigated Se speciation in commonly consumed Thai seafood prepared by different cooking methods, namely, fresh (control), boiling, frying, and grilling, using HPLC–ICP–QQQ–MS. Across all samples, Se was predominantly present in organic forms, with selenomethionine (SeMet) identified as the major species, followed by selenocystine (SeCys_2_), while inorganic forms (Se(IV) and Se(VI)) were generally below the limit of quantification. Indo-Pacific horseshoe crab (eggs) consistently presented significantly higher SeMet concentrations than all other seafood species across all cooking methods (*p* < 0.05). In addition, frying and grilling resulted in higher apparent SeMet concentrations compared to fresh and boiled samples in several species (*p* < 0.05). This increase should be interpreted as a concentration effect associated with moisture loss during high-temperature cooking, rather than a true chemical formation of SeMet. SeCys_2_ concentrations varied across species and cooking conditions, with significantly higher levels found in certain crustaceans, such as banana prawn and musk crab, particularly after boiling (*p* < 0.05). Extraction yields ranged from 77% to 94%, indicating high analytical recovery. Overall, cooking methods influenced the concentration of Se species but did not substantially alter their chemical forms. These findings suggest that commonly consumed Thai seafood is a rich source of bioavailable Se, particularly in the form of SeMet. Further research is warranted to characterize minor Se species and assess their nutritional implications.

## 1. Introduction

Selenium (Se) is an essential trace element that plays a critical role in human health through its incorporation into selenoproteins, which are involved in antioxidant defense, thyroid hormone metabolism, immune function, etc. [1,2,3]. Adequate Se intake has been associated with a reduced risk of chronic diseases, including cardiovascular disorders, certain cancers, and bone-related conditions [4]. However, both Se deficiency and excess pose significant health risks, emphasizing the importance of understanding not only total Se content in foods but also its chemical forms, or speciation, which largely determine its bioavailability, bioaccessibility, and biological activity.

Seafood is an important component of the Thai diet, particularly in coastal regions, where fish, shrimp, crabs, mollusks, and other marine products are commonly consumed as regular protein and Se sources [5], particularly in coastal populations such as those in Chonburi, Thailand, where marine products constitute a substantial proportion of daily dietary intake [6]. Commonly consumed Thai seafood species—including crustaceans, mollusks, and fish—have been reported to contain relatively high Se concentrations [7]. Importantly, Se in seafood exists in multiple chemical forms, including organic species such as selenomethionine (SeMet) and selenocystine (SeCys_2_), as well as inorganic forms such as selenite (Se (IV)) and selenate (Se (VI)) [8]. Among these, organic Se species, particularly SeMet, are generally more bioavailable (60–84%) and nutritionally beneficial, whereas inorganic forms may exhibit different absorption efficiencies and potential toxicological implications at elevated levels [9]. Despite the recognized nutritional importance of Se, most previous studies have primarily focused on total Se content rather than its speciation [10,11,12]. This represents a critical limitation, as total Se measurements do not provide sufficient insight into the nutritional quality or functional impact of Se in foods. Furthermore, Se speciation can be significantly influenced by food processing and cooking methods, which may induce chemical transformations, degradation, or redistribution of Se species [13]. Thermal treatments such as boiling, frying, and grilling—commonly employed in Thai culinary practices—can alter the chemical structure of Se compounds through mechanisms such as oxidation, volatilization, and interaction with other food matrix components. Emerging evidence suggests that cooking processes may not only affect the total Se content but also modify the relative proportions of individual Se species, thereby influencing their nutritional value and bioaccessibility [14]. For instance, high-temperature cooking methods may lead to the degradation of heat-labile organic Se compounds or promote the conversion between different species [15]. In addition, factors such as moisture loss, lipid interaction (particularly in frying), and protein denaturation may further affect Se stability and distribution within the edible portion of seafood [16]. However, comprehensive data on Se speciation in seafood subjected to different cooking methods remain limited, particularly in the context of Southeast Asian diets.

Advanced analytical techniques, such as high-performance liquid chromatography coupled with inductively coupled plasma tandem mass spectrometry (HPLC–ICP-MS/MS), have enabled more precise identification and quantification of individual Se species in complex food matrices [17,18]. These methods provide critical insights into the transformation behavior of Se during food processing and allow for a more accurate assessment of its nutritional implications. However, a systematic investigation is lacking integrating Se speciation with commonly consumed seafood species and typical cooking practices in Thailand.

Previous studies from our research group have investigated Se composition in aquatic food products subjected to thermal processing. Singhato et al. [7,8] characterized Se species in selected freshwater and marine fish and evaluated the effects of different cooking methods on Se speciation. More recently, Rueangsri et al. [19] examined total Se concentrations, moisture loss, edible portions, yield factors, and true retention across a wider range of Thai seafood species. However, information regarding Se speciation in commonly consumed Thai seafood beyond fish species remains limited. In particular, the effects of boiling, frying, and grilling on Se species distribution in crustaceans, mollusks, cephalopods, and Indo-Pacific horseshoe crab eggs have not been systematically investigated. Since the nutritional value, bioavailability, and biological functions of Se depend strongly on its chemical form, further characterization of Se species in diverse seafood matrices is warranted. Therefore, the present study extends previous work by investigating Se speciation in commonly consumed Thai seafood using HPLC–ICP–QQQ–MS and evaluating the influence of boiling, frying, and grilling on individual Se species. By characterizing the distribution of Se species across various seafood categories and culinary treatments, this study seeks to provide a more comprehensive understanding of the nutritional quality of Se in seafood.

## 2. Materials and Methods

### 2.1. Chemicals and Reagents Used in the Study

Protease from *Streptomyces griseus* Type XIV, purchased from Sigma-Aldrich (St. Louis, MO, USA), was used for enzymatic extraction. The Se species standards, including selenomethionine (SeMet), seleno-L-cystine, sodium selenate [Se(VI)], and sodium selenite [Se(IV)], were also obtained from Sigma-Aldrich (USA) and used to identify and determine the chromatographic profiles of individual Se species using HPLC. The HPLC mobile phase was prepared using ammonium phosphate and ammonium acetate, which were obtained from Merck (KGaA, Darmstadt, Germany). The accuracy of Se speciation analysis was verified using a certified reference material, SELM-1 (Se-enriched yeast), purchased from the National Research Council of Canada. Nitric acid (Suprapur^®^, 65% HNO_3_), purchased from Sigma-Aldrich (St. Louis, MO, USA), was diluted to 10% (*v*/*v*) and used for soaking all volumetric flasks and glassware prior to use. All soaked glassware was subsequently rinsed 3–4 times with deionized water obtained from a Milli-Q^®^ water purification system (MilliporeSigma, Burlington, MA, USA) and dried before use. In addition, Milli-Q^®^ deionized water was used throughout the study.

### 2.2. Seafood Sample Preparation

The seafood species included in this study were selected based on their previously reported high Se concentrations, common consumption in Thailand, and availability in local seafood markets. These species also represent economically important seafood products commonly consumed in coastal regions. The ten seafood species with the highest Se concentrations, as listed in Table 1, were selected for the determination of Se chemical forms in this study under four conditions: fresh, boiled, fried, and grilled. The locations of the markets where the samples were purchased, as well as the sample preparation protocols, including cooking procedures, homogenization, freeze-drying, and sample storage until analysis, have been described elsewhere [19].

### 2.3. Speciation Analysis of Se in Seafood Samples

#### 2.3.1. Enzymatic Extraction Procedures

Each homogenized seafood sample from each sampling location was processed independently in triplicate. The extraction procedure was adapted from Sele et al. [20] with slight modifications. Approximately 0.2 g of each sample was accurately weighed and transferred into a 50 mL volumetric vial. After that, 2.5 mL of 1 mM ammonium phosphate buffer (pH 7.5), containing 20 mg of protease Type XIV, was added, and the mixture was vortexed for 1–2 min to ensure proper dispersion. The samples were then incubated at 37 °C for 24 h in a shaking incubator (Thermo Scientific MaxQ 420, Thermo Fisher Scientific Inc., Waltham, MA, USA) at 100 rpm. To minimize photo-degradation, all vials were wrapped in aluminum foil and maintained in the dark during incubation. Following incubation, samples were allowed to equilibrate at room temperature for approximately 10 min prior to centrifugation at 10,000 rpm for 10 min. The resulting supernatants were carefully collected and filtered through 0.2 µm nylon membrane filters before instrumental analysis. Procedural blanks were prepared in parallel by adding only ammonium phosphate buffer (pH 7.5) and protease to separate vials, followed by identical extraction procedures, to monitor potential contamination. All prepared extracts, including blanks, were subsequently subjected to HPLC–ICP–QQQ–MS analysis.

#### 2.3.2. The Standard Solution of Se Species and Calibration Curve

**Figure 1:**
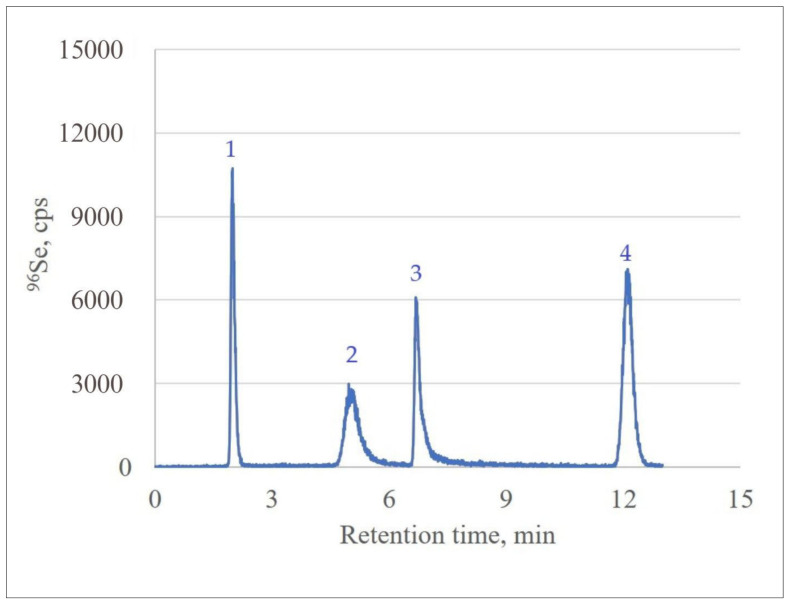
The chromatogram of a standard mixture with concentration 200 µg/L: SeCys_2_ (1), SeMet (2), Se(IV) (3), and Se(VI) (4).

Individual Se species standards, selenomethionine (SeMet), selenocystine (SeCys_2_), selenate [Se(VI)], and selenite [Se(IV)] were prepared from stock solutions to establish retention times and corresponding peak areas for chromatographic identification. A combined working standard containing all four Se species was subsequently prepared and used to generate calibration curves for quantitative analysis throughout the study. The chromatographic separation profile of the mixed standard solution (200 µg/L), obtained using anion-exchange HPLC coupled with ICP–QQQ–MS, is presented in Figure 1.

#### 2.3.3. Quality Control and Method Validations

The analytical validation parameters, including calibration performance, limits of detection (LOD), limits of quantification (LOQ), and analytical precision, were further evaluated to confirm the reliability of the Se speciation method for complex seafood matrices. The LOD for all Se species were estimated based on the criterion of three times the standard error of the regression divided by the slope (3 × SE/slope), yielding a value of 20 µg/L. Correspondingly, the LOQ were established at 50 µg/L. Method performance at the LOQ level demonstrated satisfactory recovery, with values ranging from 93.0% to 101.5% across all Se species. Precision, expressed as relative standard deviation (RSD), varied between 1.8% and 5.9%, meeting the acceptable validation criteria described in previous studies [21]. At the method level, the LOD and LOQ were determined to be 1 µg/100 g and 2 µg/100 g of sample, respectively.

Analytical accuracy was further verified using the CRM, SELM-1 (Se-enriched yeast). The reference material was prepared by the same extraction protocol as the samples and analyzed in triplicate. The resulting extracts were incorporated into the HPLC–ICP–QQQ–MS analytical sequence for quantification of SeMet. The measured SeMet concentration in SELM-1, expressed on a dry matter basis after moisture correction, was 3161.6 ± 42.1 mg/kg. This value stays within the certified reference range of 2930–3450 mg/kg, thereby confirming the reliability and accuracy of the Se speciation method.

The accuracy and completeness of Se speciation and extraction were further assessed using a mass balance approach. This evaluation aimed to verify the efficiency of the extraction procedure and the reliability of Se species quantification. Following extraction, both the residual solid fractions from the seafood samples and the corresponding liquid extracts used for HPLC–ICP–QQQ–MS analysis—including procedural blanks and SELM-1—were subjected to microwave-assisted digestion. Total Se concentrations in these digested fractions were then determined under the same analytical conditions as previously described [19]. Mass balance recovery was calculated by combining the total Se quantified in the extract and the residual fraction for each sample. Notably, Se concentrations identified in all residual fractions were below the LOQ, indicating minimal loss of analyte during the extraction process. The overall recovery, expressed as the percentage of total Se accounted for relative to previously reported total Se values [19], ranged from 90.5% to 108.6% across all samples. These values fall within acceptable analytical limits (80–120%), thereby confirming the high efficiency and reliability of the extraction and speciation procedures.

#### 2.3.4. Selenium Speciation Analysis in Seafood Samples

The Se speciation in seafood samples subjected to different cooking methods was determined following enzymatic extraction, using high-performance liquid chromatography (HPLC; 1260 Infinity II Prime LC System, Agilent Technologies, Santa Clara, CA, USA) combined with inductively coupled plasma triple quadrupole mass spectrometry (ICP–QQQ–MS; Agilent 8800, Agilent Technologies, Santa Clara, CA, USA). All analyses were conducted at the National Institute of Metrology, Thailand. Chromatographic separation of Se species was achieved using an anion-exchange column (Hamilton PRP-X100, Hamilton Company, Reno, NV, USA). The mobile phase system consisted of ammonium acetate buffers (0.5 mM and 100 mM, both adjusted to pH 5.2), applied under gradient elution conditions. A sample injection volume of 25 µL was employed, with a constant flow rate of 1.0 mL min^−1^. Detailed gradient profiles and instrumental operating parameters for the HPLC–ICP–QQQ–MS system are summarized in Supplementary Table S1.

#### 2.3.5. Determination of Total Selenium, True Retention, and Moisture Content

The seafood samples analyzed in the present study correspond to the same freeze-dried samples and species previously investigated for total Se content and true retention under several cooking conditions. Accordingly, the analytical procedures for total Se quantification, along with the associated findings on Se concentrations across cooking methods, true retention values, and moisture content, have been documented in detail in an earlier publication [19]. In addition, parameters including edible portion and yield factors for each seafood species were established in that study and were adopted in the current work.

#### 2.3.6. Statistical Analysis

Concentrations of individual Se species in seafood samples prepared under different cooking conditions (fresh, boiled, fried, and grilled), together with extraction efficiencies, were expressed as mean ± standard deviation (SD). Prior to parametric analysis, data distribution normality and homogeneity of variance were evaluated. Multiple comparisons among groups were subsequently performed using Tukey’s HSD post hoc test following two-way ANOVA. When significant effects were identified (*p* < 0.05), post hoc comparisons were conducted using Tukey’s Honestly Significant Difference (HSD) test to examine pairwise differences between groups. All statistical analyses were carried out using IBM^®^ SPSS Statistics for Windows, version 26.0.

## 3. Results

**Figure 2:**
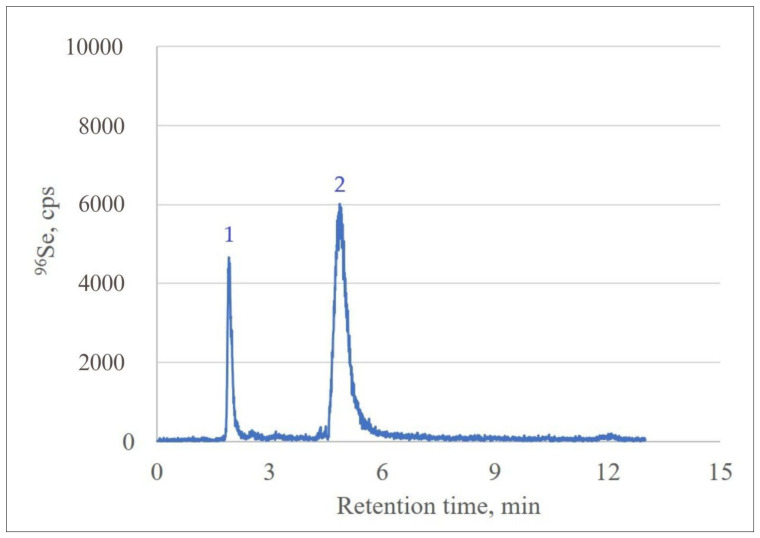
Example of the chromatogram of fresh boiled Banana prawn; SeCys_2_ (1) and SeMet (2).

Across the analyzed seafood species, Se speciation profiles revealed that the inorganic forms, Se(VI) and Se(IV), were generally below the LOQ in most sample conditions, including fresh, boiled, fried, and grilled preparations. Furthermore, mass balance evaluation confirmed the effectiveness of the extraction procedure, indicating that the majority of Se present in the samples was successfully recovered in the extractable fraction. Based on these findings, the Se composition of the seafood samples was predominantly characterized by organic species, with SeMet and SeCys_2_ identified as the principal forms (Figure 2).

### 3.1. Selenium Speciation in Seafood Under Different Cooking Methods

#### 3.1.1. Fresh Samples

In fresh seafood samples, SeMet was identified as the predominant Se chemical form across all examined species, followed by SeCys_2_. The highest SeMet concentration was found in Indo-Pacific horseshoe crab eggs, which was significantly greater than in all other seafood species (*p* < 0.05). SeCys_2_ was present at lower concentrations compared to SeMet but still contributed notably to total organic Se, particularly in crustaceans such as blue crab and musk crab. In contrast, inorganic Se species (Se(IV) and Se(VI)) were generally below the LOQ in most samples, with only minor detectable levels in selected species such as serrated mud crab, oysters, and horseshoe crab eggs. Extraction yields ranged from approximately 79.5% to 94.5%, indicating high extraction efficiency across all seafood types (Table 2).

#### 3.1.2. Boiled Samples

Following boiling, SeMet remained the dominant Se species in most seafood samples; however, a relative increase in SeCys_2_ was found in certain species, particularly banana prawn and musk crab. Inorganic Se species were still largely below LOQ, although trace amounts of Se(VI) were identified in some samples. Notably, wedge shell and horseshoe crab eggs presented significantly higher SeMet concentrations compared to other species (*p* < 0.05). Extraction yields remained high (80.5–93.0%), suggesting that the boiling process did not substantially reduce the efficiency of Se extraction (Table 3).

#### 3.1.3. Fried Sample

Frying resulted in an apparent increase in SeMet concentrations across several seafood species, with the highest levels again observed in horseshoe crab eggs, followed by wedge shell and blue crab (*p* < 0.05). SeCys_2_ levels were variable, with relatively higher concentrations in crustaceans such as banana prawn and musk crab. In contrast, inorganic Se species remained negligible in most samples, although detectable levels of Se(VI) were observed in selected species such as blue crab and oysters. Extraction yields ranged from approximately 79.0% to 93.5%, indicating consistent extraction performance despite the high-temperature cooking process (Table 4).

#### 3.1.4. Grilled Sample

In grilled seafood, SeMet continued to be the predominant Se species form across all samples, with significantly higher concentrations observed in horseshoe crab eggs and wedge shell (*p* < 0.05). SeCys_2_ was present at moderate levels, with the highest concentrations identified in musk crab. Similar to other cooking methods, inorganic Se species were generally below LOQ, with only trace levels identified in a few species. Extraction yields ranged from approximately 77.5% to 93.5%, demonstrating that grilling did not substantially compromise Se recovery (Table 5).

### 3.2. Combined Effects of Different Cooking Methods and Seafood Species on Se Species Forms

**Figure 3:**
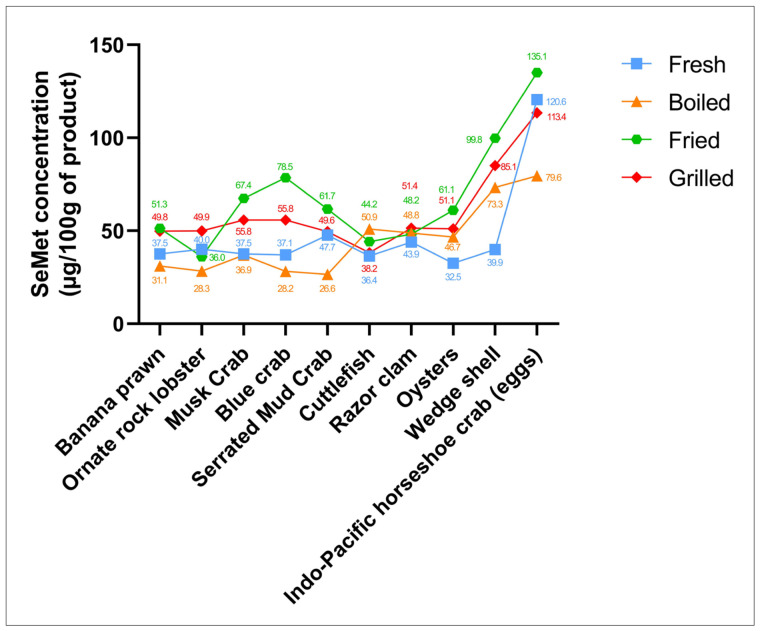
Combined effects of different seafood species and cooking methods on SeMet as Se.

The interaction between seafood species and cooking methods demonstrated a clear variation in SeMet concentrations. Overall, SeMet remained the dominant Se species across all cooking conditions; however, its levels were strongly influenced by both species type and thermal processing. Frying and grilling generally resulted in higher SeMet concentrations compared to fresh and boiled conditions, particularly in species such as wedge shell and Indo-Pacific horseshoe crab eggs. This trend suggests a concentration effect, likely due to moisture loss during high-temperature cooking. In contrast, boiling tended to produce lower SeMet values in several species, potentially reflecting leaching into the cooking medium. Species-specific differences were also evident. Indo-Pacific horseshoe crab eggs consistently exhibited the highest SeMet levels across all cooking methods, followed by wedge shell and certain crustaceans. Meanwhile, species such as oysters and cuttlefish showed comparatively lower SeMet concentrations. These findings indicate a significant interaction effect (*p* < 0.05), where the influence of cooking method on SeMet is dependent on the intrinsic composition of each seafood species (Figure 3).

**Figure 4:**
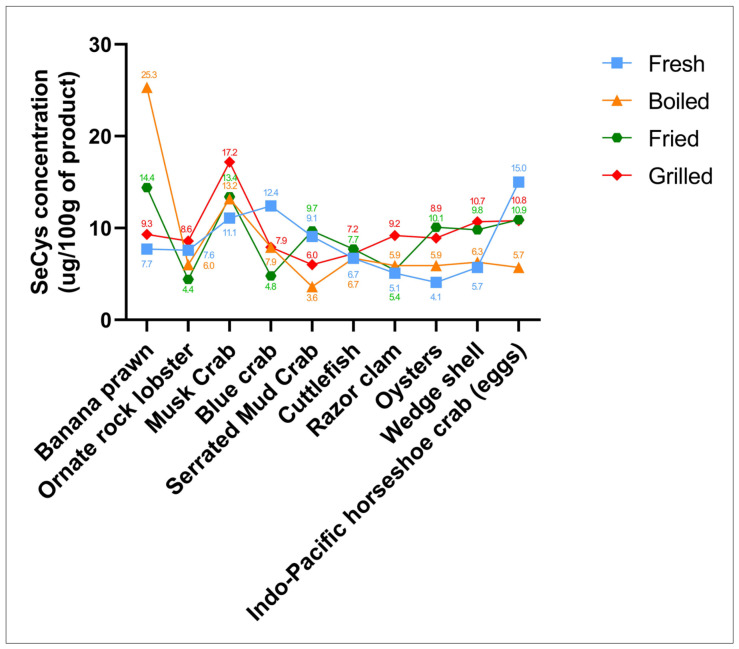
Combined effects of different seafood species and cooking methods on SeCys_2_ as Se.

In contrast to SeMet, SeCys_2_ exhibited a more variable and less consistent pattern across cooking methods. While SeCys_2_ was present in all seafood species, its concentration did not follow a uniform trend across thermal treatments. Boiling appeared to increase SeCys_2_ levels in certain species, particularly banana prawn, suggesting possible protein denaturation and release of bound Se forms. Frying and grilling showed moderate increases in some species but not consistently across all samples. Compared to SeMet, SeCys_2_ contributed a smaller proportion of total Se species and appeared more sensitive to species-specific biochemical composition rather than the cooking method alone (Figure 4).

## 4. Discussion

Previous studies from our research group have investigated Se composition in aquatic foods from different perspectives. Earlier work on seafood primarily focused on total Se concentrations, moisture loss, and retention characteristics during cooking [19], whereas Se speciation studies were limited to selected freshwater and marine fish species [8]. The present study extends this body of knowledge by examining Se speciation in a broader range of commonly consumed Thai seafood, including crustaceans, mollusks, and cephalopods. In addition, the effects of grilling, alongside boiling and frying, were evaluated. The results demonstrate that thermal processing affects individual Se species differently. In particular, SeMet remained the dominant and relatively stable Se species across cooking methods, whereas SeCys_2_ exhibited greater variability, suggesting a more complex response to thermal processing and seafood matrix composition. The findings consistently demonstrated that inorganic Se species, Se(IV) and Se(VI), were predominantly below the LOQ across all sample conditions [22]. The LOQ values for Se(IV) and Se(VI) were 50 µg/L under the applied analytical conditions. The low detectability of inorganic Se species may also partly reflect matrix-related signal suppression associated with complex seafood extracts. These findings align with previous reports indicating that inorganic Se forms are generally present at low levels in animal-derived foods [23,24,25], as they are rapidly metabolized into organic forms within biological systems [26,27]. The predominance of organic Se species, particularly SeMet and SeCys_2_, observed in this study is consistent with established biochemical pathways of Se metabolism [28]. In both marine organisms and terrestrial animals, inorganic Se obtained from the environment or feed is efficiently converted into organic forms, especially SeMet, which can be nonspecifically incorporated into proteins in place of methionine [29]. This metabolic characteristic explains why SeMet was consistently identified as the dominant Se species across all seafood types and cooking conditions. The observed distribution of Se species can be explained by underlying biochemical and physicochemical mechanisms. The predominance of SeMet observed in the analyzed seafood species is likely related to its biological incorporation into proteins in place of methionine, resulting in greater accumulation and stability compared to other Se species. Species-specific metabolism and trophic characteristics may additionally influence Se accumulation and speciation profiles among marine organisms. The observed variations in SeCys_2_ concentrations following thermal processing may be associated with protein denaturation, oxidative transformation, and possible interconversion among Se-containing compounds during heating [30]. Additionally, SeCys_2_ is considered chemically less stable than SeMet and may be more susceptible to oxidation and degradation under high-temperature conditions [31].

It should be noted that Se concentrations in the present study were expressed on a wet weight basis to reflect actual consumer intake conditions. Therefore, increases observed after frying and grilling should be interpreted primarily as concentration effects associated with moisture loss during thermal processing rather than true increases in Se content. During frying, lipid oxidation products generated under high-temperature conditions may interact with Se compounds and influence their chemical stability, extractability, and apparent concentrations [32]. In contrast, boiling often resulted in comparatively lower SeMet levels, likely due to leaching of water-soluble Se compounds into the cooking medium [33]. Similar results have been reported in previous studies, where water-based cooking methods led to nutrient losses, whereas dry-heat methods concentrated nutrients [34]. In contrast to SeMet, SeCys_2_ exhibited a more variable response to cooking processes. In some species, particularly crustaceans such as banana prawn and musk crab, boiling resulted in increased SeCys_2_ concentrations. This may be attributed to protein denaturation, which enhances the release of bound Se species into extractable forms [35]. However, SeCys_2_ is known to be chemically less stable than SeMet, being susceptible to oxidation, heat degradation, and pH variations [36]. These properties likely contribute to its inconsistent behavior across cooking methods observed in this study. Another plausible explanation for changes in Se species distribution is the interconversion between Se compounds during heat treatment and the potential for oxidation artifacts during sample preparation. The enzymatic extraction involved incubation at 37 °C for 24 h, which may promote oxidative transformations of Se species. For example, selenocysteine can be oxidized to selenocystine or further transformed into other oxidized Se forms. Therefore, part of the observed SeCys_2_ may reflect both native compounds and artifacts generated during extraction. This possibility should be taken into account when interpreting speciation results [37].

A single enzymatic extraction protocol was applied uniformly across all seafood matrices to ensure analytical consistency and comparability between species and cooking conditions [38]. The comparatively lower recoveries observed in certain samples may be associated with incomplete extraction of tightly protein-bound Se species, matrix complexity, or partial oxidative and thermal transformation of Se compounds during sample preparation. As a result, non-extractable Se fractions, including tightly bound or insoluble species, as well as potential lipid-associated Se compounds, may not be fully recovered. This limitation is particularly relevant for seafood matrices, where lipid-soluble Se compounds may contribute to the total Se pool. In this study, the consistently high extraction yields (approximately 77–94%) across all seafood species and cooking methods further support the robustness of the analytical method used in this study. Nevertheless, the fact that the sum of identified Se species did not fully account for total extractable Se suggests the presence of additional Se compounds not targeted in this analysis. Although SeMet and SeCys_2_ accounted for the majority of quantified Se species, a fraction of total Se remained uncharacterized. This observation highlights a key limitation but also an important study opportunity. Previous studies have identified additional Se compounds in seafood, including selenoneine, selenomethionine-Se-oxide, and other low-molecular-weight Se metabolites [39,40,41]. In the present study, these compounds were not directly confirmed due to the absence of specific standards or spiking experiments. Therefore, it is likely that a portion of Se exists in unidentified or co-eluting species. Future studies employing targeted spiking experiments and high-resolution mass spectrometry are needed to fully characterize the Se metabolome in seafood. The lack of spiking experiments for compounds such as selenoneine represents a limitation of the current study, as it prevents definitive identification of minor Se species. Consequently, the present results should be interpreted as representing the major detectable species under the applied analytical conditions.

Selenium speciation profiles in seafood may additionally vary according to environmental Se availability, geographical origin, seasonal variation, and aquaculture versus wild-caught production systems. The Se species concentrations observed in the present study were generally comparable with values previously reported in seafood species from other geographical regions, although variations may occur due to species differences, environmental conditions, and dietary exposure [42]. However, the seafood examined in this study represents commonly consumed and economically accessible species in Thailand, highlighting their potential contribution to dietary Se intake. Given that SeMet is highly bioavailable and efficiently incorporated into body proteins, the dominance of this form in Thai seafood suggests a favorable nutritional profile. From a public health perspective, the findings emphasize that both seafood type and cooking method should be considered when evaluating Se intake. While cooking does not appear to drastically alter Se speciation, it significantly affects the concentration of bioactive forms, particularly through physical changes such as moisture loss or leaching. Several seafood species analyzed in this study, particularly Indo-Pacific horseshoe crab eggs and wedge shell, contained relatively high concentrations of SeMet and may therefore represent valuable dietary Se sources. Nevertheless, the contribution of individual seafood species to dietary Se intake is influenced not only by Se concentration but also by typical consumption patterns and portion sizes. Consequently, the high SeMet concentrations observed in Indo-Pacific horseshoe crab eggs may not necessarily translate into the highest dietary Se intake. Overall, the Se concentrations identified in several seafood samples could contribute substantially to the recommended daily Se intake for adults. Therefore, the findings of this study may provide useful information for nutritional assessment, dietary planning, and the development of evidence-based recommendations regarding Se intake from seafood products. Finally, it is important to consider the broader context of seafood safety. Marine foods may contain contaminants such as mercury (Hg), arsenic (As), cadmium (Cd), and lead (Pb), which could pose health risks upon long-term consumption [43,44]. Selenium has been shown to mitigate the toxicity of certain heavy metals, particularly methylmercury, through the formation of biologically inert complexes [45]. Therefore, future studies should investigate the interaction between Se speciation and heavy metal contamination in Thai seafood to better assess both nutritional benefits and potential risks.

## 5. Conclusions

This study demonstrates that Se speciation in commonly consumed Thai seafood is predominantly characterized by organic forms, particularly SeMet, with SeCys_2_ present at lower concentrations, while inorganic species were generally below quantifiable levels. Across all cooking methods, the overall speciation profile remained relatively stable, indicating that thermal processing does not substantially alter the fundamental chemical forms of Se in seafood. However, cooking methods influenced the measured concentrations of Se species. Frying and grilling were associated with higher apparent levels of SeMet, likely due to moisture loss and subsequent concentration effects, whereas boiling tended to yield lower concentrations, possibly reflecting leaching into the cooking medium. SeCys_2_ exhibited greater variability across species and cooking conditions, suggesting sensitivity to protein denaturation and potential chemical transformation during heat processing. The consistently high extraction efficiency supports the reliability of the analytical approach, although the incomplete recovery of total Se implies the presence of additional, uncharacterized Se compounds in seafood matrices. Overall, these findings highlight that both seafood species and cooking methods influence the nutritional availability of Se, particularly in its bioactive organic forms. From a dietary perspective, commonly consumed Thai seafood represents a valuable source of bioavailable Se. Future studies should further investigate unidentified Se compounds, Se bioaccessibility, and the influence of industrial food processing on Se speciation in seafood.

## Figures and Tables

**Table 1:** The ten selected seafood species in this study.

Common Name	Scientific Name	Local Name	Purchase
			**(Month/Year)**
Shrimp and prawn (captured)			
Banana prawn	*Fenneropenaeus merguiensis*	Koong Share Buay	2/2025
Ornate rock lobster	*Panulirus ornatus*	Koong Mungkorn	2/2025
Crabs (captured)			
Musk Crab	*Charybdis feriata* Linnaeus	Pu Lai Sua	2/2025
Blue crab	*Portunus pelagicus*	Pu Ma	3/2025
Serrated Mud Crab	*Scylla serrata*	Pu Dam	3/2025
Squids (captured)			
Cuttlefish	*Sepia brevimana*	Pla Muek Kradong	3/2025
Shellfish (captured)			
Razor clam	*Solen strictus* Gould	Hoi Lord	3/2025
Oysters	*Crassostrea gigas*	Hoi Nang Rom	3/2025
Wedge shell	*Mercenaria mercenaria*	Hoi Talab	3/2025
Indo-Pacific horseshoe crab (eggs)	*Tachypleus gigas*	Mangda Jan	3/2025

**Table 2:** The marginal mean of Se species concentration in fresh seafood (*n* = 3).

Common Name	SeMet as Se (µg/100 g of Product)	SeCys_2_ as Se (µg/100 g of Product)	Se(IV) as Se (µg/100 g of Product)	Se(VI) as Se (µg/100 g of Product)	Total Se (µg/100 g of Product)	Sum of Se in Extracts (µg/100 g of Product)	Extraction Yield (%)
Banana prawn	37.5 ± 0.00 ^c,d^	7.7 ± 0.03 ^b,c^	<LOQ	<LOQ	49.5 ± 0.30	45.2 ± 0.90 ^c,d^	91.5 ± 2.11
Ornate rock lobster	40.0 ± 0.01 ^b,c^	7.6 ± 0.03 ^b,c^	<LOQ	<LOQ	57.5 ± 0.90	47.7 ± 0.31 ^c^	83.0 ± 4.22
Musk Crab	37.5 ± 0.00 ^c,d^	11.1 ± 0.08 ^a,b^	<LOQ	1.0 ± 0.00 ^a^	52.7 ± 0.16	49.8 ± 0.05 ^c^	94.5 ± 2.10
Blue crab	37.1 ± 0.02 ^c,d^	12.4 ± 0.05 ^a,b^	<LOQ	<LOQ	53.1 ± 0.90	49.6 ± 0.51 ^c^	93.5 ± 2.10
Serrated Mud Crab	47.7 ± 0.03 ^b^	9.1 ± 0.02 ^a,b,c^	1.0 ± 0.00 ^a^	1.0 ± 0.00 ^a^	71.2 ± 0.80	59.1 ± 0.08 ^b^	83.0 ± 1.43
Cuttlefish	36.4 ± 0.02 ^c,d^	6.7 ± 0.02 ^b,c^	<LOQ	<LOQ	50.8 ± 0.08	43.1 ± 0.81 ^c,d^	85.0 ± 2.82
Razor clam	43.9 ± 0.11 ^b,c^	5.1 ± 0.02 ^b,c^	<LOQ	1.4 ± 0.01 ^a^	57.8 ± 0.08	50.5 ± 0.83 ^c^	87.5 ± 4.90
Oysters	32.5 ± 0.02 ^d^	4.1 ± 0.01 ^c^	1.1 ± 0.00 ^a^	0.7 ± 0.00 ^a^	48.6 ± 0.09	38.6 ± 0.42 ^d^	79.5 ± 0.71
Wedge shell	39.9 ± 0.01 ^b,c,d^	5.7 ± 0.04 ^b,c^	<LOQ	0.8 ± 0.00 ^a^	53.5 ± 0.25	46.5 ± 0.51 ^c,d^	87.0 ± 2.82
Indo-Pacific horseshoe crab (eggs)	120.6 ± 0.01 ^a^	15.0 ± 0.06 ^a^	3.9 ± 0.00 ^a^	2.5 ± 0.01 ^a^	155.5 ± 0.85	142.2 ± 0.80 ^a^	91.5 ± 2.10

For a given variable, estimated marginal means + standard deviation from 3 individual samples with different superscript letters in the same column were significantly different (*p* < 0.05 two-way ANOVA followed by Tukey’s HSD post hoc multiple comparisons). Different superscripts in the same column indicate significant differences at *p* < 0.05. Data on total Se concentrations also appeared in a previously published study [19].

**Table 3:** The marginal mean of Se species concentration in boiled seafood (*n* = 3).

Common Name	SeMet as Se (µg/100 g of Product)	SeCys_2_ as Se (µg/100 g of Product)	Se(IV) as Se (µg/100 g of Product)	Se(VI) as Se (µg/100 g of Product)	Total Se (µg/100 g of Product)	Sum of Se in Extracts (µg/100 g of Product)	Extraction Yield (%)
Banana prawn	31.1 ± 0.00 ^c,d^	25.3 ± 0.03 ^a^	<LOQ	<LOQ	61.9 ± 0.40 ^b,c^	57.5 ± 0.71	93.0 ± 2.87
Ornate rock lobster	28.3 ± 0.01 ^d^	6.0 ± 0.01 ^c^	<LOQ	<LOQ	40.4 ± 0.06 ^c^	34.3 ± 0.42	85.0 ± 1.43
Musk Crab	36.9 ± 0.00 ^c^	13.2 ± 0.01 ^b^	<LOQ	0.9 ± 0.00 ^a^	56.9 ± 0.08 ^b,c^	51.2 ± 0.10	90.0 ± 1.40
Blue crab	28.2 ± 0.00 ^d^	7.9 ± 0.02 ^c^	<LOQ	2.9 ± 0.00 ^a^	42.3 ± 0.51 ^c^	39.1 ± 0.30	92.5 ± 0.70
Serrated Mud Crab	26.6 ± 0.01 ^d^	3.6 ± 0.00 ^c^	<LOQ	0.8 ± 0.00 ^a^	33.6 ± 0.82 ^c^	31.0 ± 0.82	92.5 ± 2.12
Cuttlefish	50.9 ± 0.00 ^b^	6.7 ± 0.00 ^c^	<LOQ	<LOQ	63.0 ± 0.94 ^b^	57.6 ± 0.53	91.5 ± 2.10
Razor clam	48.8 ± 0.02 ^b^	5.9 ± 0.04 ^c^	<LOQ	1.0 ± 0.00 ^a^	63.4 ± 0.55 ^b^	55.7 ± 0.90	88.0 ± 1.40
Oysters	46.7 ± 0.01 ^b^	5.9 ± 0.02 ^c^	1.28 ± 0.00 ^a^	1.0 ± 0.00 ^a^	68.4 ± 0.06 ^b^	55.0 ± 0.64	80.5 ± 0.72
Wedge shell	73.3 ± 0.02 ^a^	6.3 ± 0.02 ^c^	<LOQ	<LOQ	93.2 ± 0.57 ^a,b^	79.6 ± 0.95	85.5 ± 3.57
Indo-Pacific horseshoe crab (eggs)	79.6 ± 0.02 ^a^	5.7 ± 0.05 ^c^	5.74 ± 0.00 ^a^	2.1 ± 0.00 ^a^	106.6 ± 0.71 ^a^	93.2 ± 0.83	87.5 ± 0.77

For a given variable, estimated marginal means + standard deviation from 3 individual samples with different superscript letters in the same column were significantly different (*p* < 0.05 two-way ANOVA followed by Tukey’s HSD post hoc multiple comparisons). Different superscripts in the same column indicate significant difference at *p* < 0.05. Data on total Se concentrations also appeared in a previously published study [19].

**Table 4:** The marginal mean of Se species concentration in fried seafood (*n* = 3).

Common Name	SeMet as Se (µg/100 g of Product)	SeCys_2_ as Se (µg/100 g of Product)	Se(IV) as Se (µg/100 g of Product)	Se(VI) as Se (µg/100 g of Product)	Total Se (µg/100 g of Product)	Sum of Se in Extracts (µg/100 g of Product)	Extraction Yield (%)
Banana prawn	51.3 ± 0.03 ^e^	14.4 ± 0.09 ^a^	<LOQ	<LOQ	74.7 ± 0.81 ^d,e^	65.7 ± 0.43	88.0 ± 2.82
Ornate rock lobster	36.0 ± 0.03 ^g^	4.4 ± 0.00 ^b^	<LOQ	<LOQ	47.1 ± 0.50 ^f^	40.5 ± 0.10	86.0 ± 1.41
Musk Crab	67.4 ± 0.02 ^d^	13.4 ± 0.08 ^a^	<LOQ	<LOQ	87.9 ± 0.90 ^c,d^	80.8 ± 0.71	92.0 ± 1.40
Blue crab	78.5 ± 0.00 ^c^	4.8 ± 0.04 ^b^	<LOQ	6.7 ± 0.00 ^a^	97.4 ± 0.51 ^b,c^	90.1 ± 0.07	92.5 ± 0.71
Serrated Mud Crab	61.7 ± 0.04 ^d^	9.7 ± 0.07 ^a,b^	<LOQ	1.4 ± 0.00 ^a^	78.0 ± 0.20 ^d,e^	72.9 ± 0.38	93.5 ± 0.73
Cuttlefish	44.2 ± 0.00 ^f^	7.7 ± 0.05 ^a,b^	<LOQ	<LOQ	65.8 ± 0.30 ^e,f^	51.9 ± 0.83	79.0 ± 2.87
Razor clam	48.2 ± 0.01 ^e,f^	5.4 ± 0.04 ^b^	<LOQ	1.2 ± 0.00 ^a^	64.5 ± 0.41 ^e,f^	54.8 ± 0.31	85.0 ± 1.49
Oysters	61.1 ± 0.01 ^d^	10.1 ± 0.03 ^a,b^	2.0 ± 0.00 ^b^	1.2 ± 0.00 ^a^	86.7 ± 0.76 ^c,d^	74.5 ± 0.60	86.0 ± 0.06
Wedge shell	99.8 ± 0.06 ^b^	9.8 ± 0.01 ^a,b^	<LOQ	<LOQ	129.7 ± 0.09 ^b^	109.6 ± 0.06	84.5 ± 0.71
Indo-Pacific horseshoe crab (eggs)	135.1 ± 0.08 ^a^	10.9 ± 0.02 ^a,b^	10.3 ± 0.01 ^a^	4.5 ± 0.00 ^a^	193.9 ± 0.56 ^a^	160.9 ± 0.42	83.0 ± 2.81

For a given variable, estimated marginal means + standard deviation from 3 individual samples with different superscript letters in the same column were significantly different (*p* < 0.05 two-way ANOVA followed by Tukey’s HSD post hoc multiple comparisons). Different superscripts in the same column indicate significant difference at *p* < 0.05. Data on total Se concentrations also appeared in a previously published study [19].

**Table 5:** The marginal mean of Se species concentration in grilled seafood (*n* = 3).

Common Name	SeMet as Se (µg/100 g of Product)	SeCys_2_ as Se (µg/100 g of Product)	Se(IV) as Se (µg/100 g of Product)	Se(VI) as Se (µg/100 g of Product)	Total Se (µg/100 g of Product)	Sum of Se in Extracts (µg/100 g of Product)	Extraction Yield (%)
Banana prawn	49.8 ± 0.02 ^c^	9.3 ± 0.02 ^b^	<LOQ	<LOQ	66.5 ± 0.80 ^d^	59.1 ± 0.94	89.0 ± 2.80
Ornate rock lobster	49.9 ± 0.01 ^c^	8.6 ± 0.04 ^b^	<LOQ	<LOQ	64.3 ± 0.80 ^d^	58.5 ± 0.19	91.0 ± 0.09
Musk Crab	55.8 ± 0.01 ^c^	17.2 ± 0.01 ^a^	<LOQ	2.5 ± 0.00 ^a^	80.9 ± 0.18 ^c,d^	75.6 ± 0.46	93.5 ± 2.10
Blue crab	55.8 ± 0.02 ^c^	7.9 ± 0.02 ^b^	<LOQ	1.6 ± 0.01 ^a^	79.8 ± 0.96 ^c,d^	65.4 ± 0.47	82.0 ± 0.02
Serrated Mud Crab	49.6 ± 0.00 ^c^	6.0 ± 0.02 ^b^	<LOQ	1.4 ± 0.00 ^a^	73.8 ± 0.08 ^c,d^	57.2 ± 0.06	77.5 ± 0.73
Cuttlefish	38.2 ± 0.00 ^d^	7.2 ± 0.02 ^b^	<LOQ	<LOQ	51.5 ± 0.83 ^d^	45.5 ± 0.85	88.5 ± 4.93
Razor clam	51.4 ± 0.00 ^c^	9.2 ± 0.01 ^b^	<LOQ	1.3 ± 0.00 ^a^	73.4 ± 0.06 ^c,d^	62.0 ± 0.22	84.5 ± 3.57
Oysters	51.1 ± 0.00 ^c^	8.9 ± 0.00 ^b^	1.7 ± 0.00 ^b^	1.0 ± 0.00 ^a^	77.5 ± 0.07 ^c,d^	62.7 ± 0.81	81.0 ± 1.42
Wedge shell	85.1 ± 0.03 ^b^	10.7 ± 0.00 ^b^	<LOQ	<LOQ	108.4 ± 0.15 ^b,c^	95.9 ± 0.33	88.5 ± 2.17
Indo-Pacific horseshoe crab (eggs)	113.4 ± 0.02 ^a^	10.8 ± 0.00 ^b^	9.8 ± 0.01 ^a^	2.8 ± 0.00 ^a^	160.1 ± 0.16 ^a^	136.8 ± 0.93	85.5 ± 2.15

For a given variable, estimated marginal means + standard deviation from 3 individual samples with different superscript letters in the same column were significantly different (*p* < 0.05 two-way ANOVA followed by Tukey’s HSD post hoc multiple comparisons). Different superscripts in the same column indicate significant difference at *p* < 0.05. Data on total Se concentrations also appeared in a previously published study [19].

## Data Availability

The original contributions presented in this study are included in the article/Supplementary Material. Further inquiries can be directed to the corresponding author.

## Supplementary Materials

The following supporting information can be downloaded at: https://www.mdpi.com/article/10.3390/foods15122052/s1, Table S1: Parameters used in analysis using HPLC-ICP-QQQ-MS.

## Abbreviations

The following abbreviations are used in this manuscript:

ANOVA	Analysis of variance
°C	Degree Celsius
CA	California
CRMs	Certified reference materials
DI	Deionized
EP	Edible portion
FAO	Food and Agriculture Organization
g	Gram
He	Helium
Hg	Mercury
HNO_3_	Nitric acid
HPLC	High-Performance Liquid Chromatography
HSD	Honestly significant difference
IBM	International Business Machines Corporation
ICP-QQQ-MS	Inductively coupled plasma triple quadrupole mass spectrometry
kg	Kilogram
L	Littre
LOQ	Limit of Quantification
LOD	Limit of Detection
M	Molar
MeHG	Methylmercury
mL	Millilitre
*n*	Number
NaHCO_3_	Sodium bicarbonate
Pb	Lead
r	Correlation coefficients
Se	Selenium
SeCyS_2_	Selenocystine
SeMet	Selenomethionine
Se(IV)	Selenite
Se(VI)	Selenate
SD	Standard deviation
SPSS	Statistical Package for the Social Sciences
USA	United States of America
*v*/*v*	Volume by volume
µg	Micro gram
µm	Micrometre
%	Percent
