# Supplementary material for: Selenium Speciation in Commonly Consumed Thai Seafood Under Different Cooking Methods
Source: Foods. 2026 Jun 6;15(12):2052. doi: 10.3390/foods15122052 (PMC13298069; doi:10.3390/foods15122052)
Supplement: Supplementary file 1 [file foods-15-02052-s001.zip › foods-4335518-supplementary.pdf]

Table S1. Parameters used in analysis using HPLC-ICP-QQQ-MS

**HPLC Parameters:**	
Column	Hamilton anion PRP-X100, 10 μm, 250 x 4.1 mm
Mobile Phase A	0.5 mM ammonium acetate, pH 5.2
Mobile Phase B	100 mM ammonium acetate, pH 5.2
Injection volume	25 μL
Flow rate	1.0 mL min^{-1}
Separation Mode	Gradient
	0-2 min: 10% B
	2-8 min: 10-100% B
	8-17 min: 100% B
	17.1-22 min: 10% B
**ICP-QQQ-MS Parameters:**	
RF power	1550 W
Sampling depth	8.0 mm
Carrier gas flow rate	1.18 L min^{-1}
Nebulizer pump	0.10 rps
Nebulizer type	Meinhard
Spray chamber	1 °C
Skimmer cone	Nickel
Sampling cone	Nickel
Reaction cell O_2 flow	30 %
m/z and Isotopes monitored	^{78}Se as $^{78}Se^{16}O^+$ on m/z 94, ^{80}Se as $^{80}Se^{16}O^+$ on m/z 96, ^{82}Se as $^{82}Se^{16}O^+$ on m/z 98
